# ATRAP Expression in Brown Adipose Tissue Does Not Influence the Development of Diet-Induced Metabolic Disorders in Mice

**DOI:** 10.3390/ijms18030676

**Published:** 2017-03-21

**Authors:** Kohji Ohki, Hiromichi Wakui, Kengo Azushima, Kazushi Uneda, Sona Haku, Ryu Kobayashi, Kotaro Haruhara, Sho Kinguchi, Miyuki Matsuda, Masato Ohsawa, Akinobu Maeda, Shintaro Minegishi, Tomoaki Ishigami, Yoshiyuki Toya, Akio Yamashita, Satoshi Umemura, Kouichi Tamura

**Affiliations:** 1 Department of Medical Science and Cardiorenal Medicine, Yokohama City University Graduate School of Medicine, 3–9 Fukuura, Kanazawa-ku, Yokohama 236-0004, Japan; t146017e@yokohama-cu.ac.jp (K.O.); k_uneda@yokohama-cu.ac.jp (K.U.); t106048f@yokohama-cu.ac.jp (S.H.); t136034d@yokohama-cu.ac.jp (R.K.); kspring00712@gmail.com (K.H.); ja472t@bma.biglobe.ne.jp (S.K.); mmatsuda@yokohama-cu.ac.jp (M.M.); o.masato@gmail.com (M.O.); maeda_chigasaki@yahoo.co.jp (A.M.); minegish@yokohama-cu.ac.jp (S.M.); tommmish@yokohama-cu.ac.jp (T.I.); ystoya@yokohama-cu.ac.jp (Y.T.); umemuras@med.yokohama-cu.ac.jp (S.U.); 2 Cardiovascular and Metabolic Disorders Program, Duke-NUS Medical School, 8 College Road, Singapore 169857, Singapore; 3 Department of Molecular Biology, Yokohama City University Graduate School of Medicine, 3–9 Fukuura, Kanazawa-ku, Yokohama 236-0004, Japan; yamasita@yokohama-cu.ac.jp; 4 Yokohama Rosai Hospital, 3211 Kozukue-cho, Kohoku-ku, Yokohama 222-0036, Japan

**Keywords:** Cre/loxP, angiotensin, receptor, adipocyte, obesity

## Abstract

Activation of tissue renin–angiotensin system (RAS), mainly mediated by an angiotensin II (Ang II) type 1 receptor (AT1R), plays an important role in the development of obesity-related metabolic disorders. We have shown that AT1R-associated protein (ATRAP), a specific binding protein of AT1R, functions as an endogenous inhibitor to prevent excessive activation of tissue RAS. In the present study, we newly generated ATRAP/*Agtrap*-floxed (ATRAP^fl/fl^) mice and adipose tissue-specific ATRAP downregulated (ATRAP^adipoq^) mice by the Cre/loxP system using *Adipoq-Cre*. Using these mice, we examined the functional role of adipose ATRAP in the pathogenesis of obesity-related metabolic disorders. Compared with ATRAP^fl/fl^ mice, ATRAP^adipoq^ mice exhibited a decreased ATRAP expression in visceral white adipose tissue (WAT) and brown adipose tissue (BAT) by approximately 30% and 85%, respectively. When mice were fed a high-fat diet, ATRAP^fl/fl^ mice showed decreased endogenous ATRAP expression in WAT that was equivalent to ATRAP^adipoq^ mice, and there was no difference in the exacerbation of dietary obesity and glucose and lipid metabolism. These results indicate that ATRAP in BAT does not influence the pathogenesis of dietary obesity or metabolic disorders. Future studies that modulate ATRAP in WAT are necessary to assess its in vivo functions in the development of obesity-related metabolic disorders.

## 1. Introduction

As the number of overweight and obese patients continues to increase worldwide, studies on the resulting health risks have gained significant attention [1,2]. Particularly, patients with excess visceral fat, such as those with metabolic syndrome, are at risk of developing a cardiovascular disease because of the acceleration of dyslipidemia and insulin resistance, as well as hypertension and type-2 diabetes mellitus [3,4,5,6]. Recent studies have focused on the pathological molecular mechanisms by which chronic adipose tissue inflammation and dysregulation of adipokines contribute to systemic insulin resistance in the progression of visceral fat obesity [7,8,9]. Previous studies demonstrated that the renin–angiotensin system (RAS), which is mainly mediated by the angiotensin II (Ang II) type-1 receptor (AT1R), plays an important role in obesity [10,11,12]. Angiotensin II type-1 receptor is involved in the regulation of physiological functions, including glucose metabolism, lipid metabolism, and adipogenesis [13]. Furthermore, the excessive activation of AT1R signaling increases oxidative stress and chronic inflammation, which results in the induction of insulin resistance. We have shown that AT1R-associated protein (ATRAP) directly binds to the C-terminus of AT1R. Angiotensin II type-1 receptor-associated protein promotes the constitutive internalization of AT1R, and is therefore likely to be an endogenous inhibitor that selectively prevents excessive activation of the AT1R signaling pathway. Angiotensin II type-1 receptor-associated protein and AT1R are endogenously expressed in many organs. Notably, adipose tissue shows high endogenous expression levels of ATRAP [14,15,16,17]. In our previous study, systemic ATRAP-knockout mice fed a high-fat diet (HFD) showed increased significant visceral fat, adipocyte hypertrophy, enhanced inflammation with macrophage infiltration of adipose tissue, and exacerbation of systemic insulin resistance compared with wild-type mice fed an HFD. Moreover, the transplantation of epididymal white adipose tissue (WAT), which highly expresses exogenous ATRAP, to systemic ATRAP knockout mice, caused a reduction in visceral fat and insulin resistance [16].

Here, we generated mice with suppressed adipose tissue-specific ATRAP expression to examine the functional role of adipose ATRAP in the pathogenesis of obesity-related metabolic disorders. To suppress ATRAP expression specifically in adipose tissue, we used mice expressing Cre recombinase with Cre/loxP system, which is driven by the adiponectin gene promoter [18]. When we generated mice where the ATRAP gene was recombined in adipocytes (ATRAP^adipoq^ mice), they showed decreased ATRAP expression in the WAT and brown adipose tissue (BAT) by approximately 30% and 85%, respectively, compared with control *Agtrap*-floxed (ATRAP^fl/fl^) mice. ATRAP expression did not change in other analyzed organs. ATRAP^fl/fl^ mice fed an HFD showed decreased endogenous ATRAP expression in the WAT that was equivalent to ATRAP^adipoq^ mice fed an HFD. No difference in the exacerbation of dietary obesity, and glucose and lipid metabolism disorders mediated by an HFD was observed between genotypes. These results suggest that ATRAP expression in BAT did not influence the pathogenesis of obesity-related metabolic disorders. Future studies that modulate ATRAP in WAT are necessary to assess the in vivo functions of ATRAP in WAT.

## 2. Results

### 2.1. Generation of Adipose Tissue-Specific ATRAP Downregulated Mice

**Figure 1:**
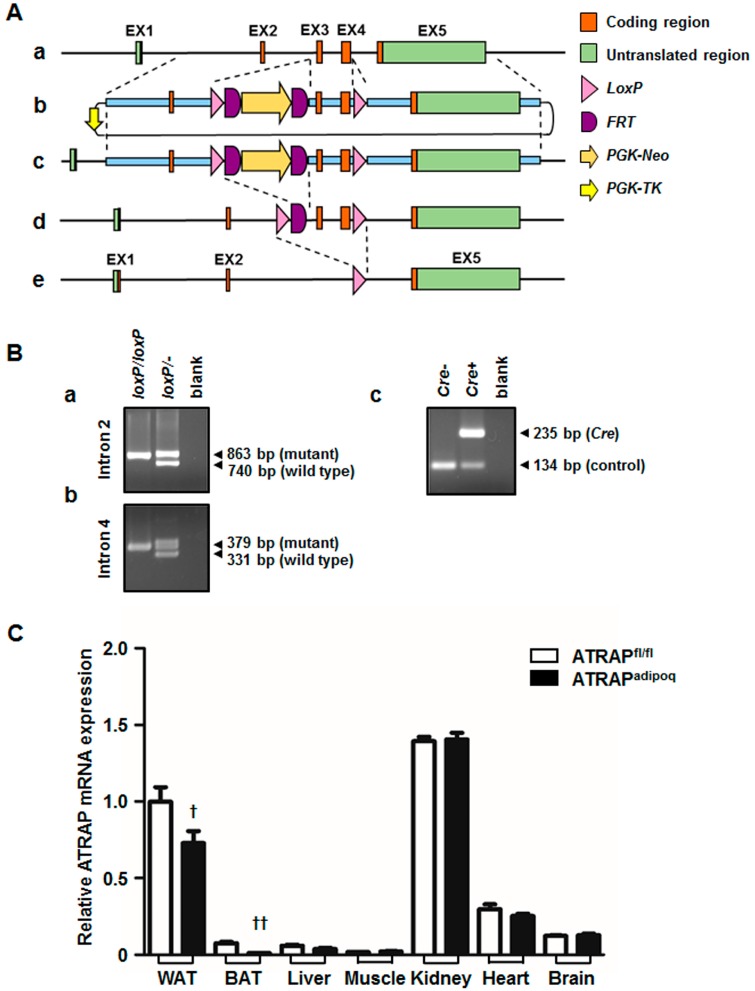

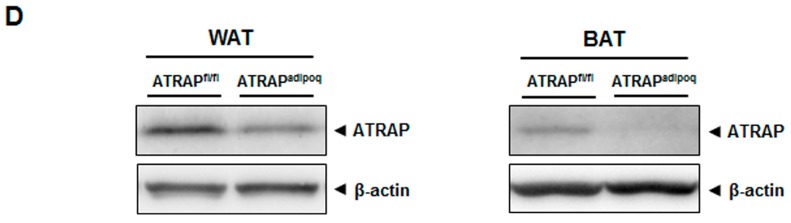
Generation of adipose tissue-specific angiotensin II type-1 receptor-associated protein (ATRAP) downregulated mice. (**A**) Schematic representation of *Adipoq-Cre* mediated recombination in adipocytes: (**a**) wild-type allele; (**b**) targeting vector; (**c**) targeted allele; (**d**) Flp recombinase-mediated allele; and (**e**) *Cre*-mediated allele. (**B**) Agarose-gel electrophoresis of PCR products amplified with following primer sets, (**a**) F23005 and R23744, (**b**) F24587 and R24917, and (**c**) Cre-fw and Cre-rw for *Cre* transgene, mAgtrapChIPF and mAgtrapChIPR for *Agtrap* gene as an internal control. These primer sequences are described in Table 1. (**C**) The relative ATRAP mRNA levels in each tissue (WAT, BAT, liver, muscle, kidney, heart, and brain) of ATRAP^adipoq^ and ATRAP^fl/fl^ mice aged 19–24 weeks on an LFD (*n* = 3–10). (**D**) Representative Western blots of ATRAP protein expression in WAT and BAT of ATRAP^fl/fl^ mice on an LFD. Values are the means ± standard error of the mean (SEM). ^†^ *p* < 0.05, ^††^ *p* < 0.01 vs. ATRAP^fl/fl^ mice. Data were analyzed by unpaired Student’s *t*-test. EX: Exon; *PGK-Neo*: Phosphoglycerate kinase 1 promoter driven neomycin resistant gene; *PGK-TK*: Phosphoglycerate kinase 1 promoter driven SV40 thymidine kinase gene; WAT: White adipose tissue; BAT: Brown adipose tissue; LFD: Low-fat diet.

**Figure 2:**
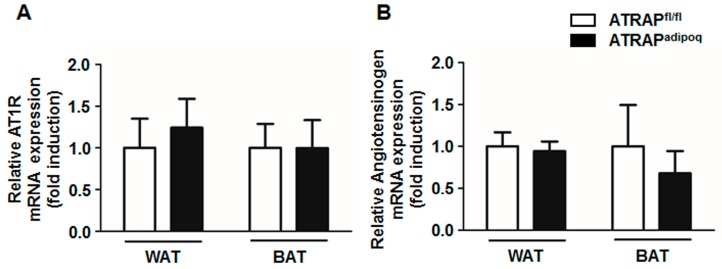
The relative expression of renin–angiotensin system (RAS) components in adipose tissues. Relative angiotensin II type-1 receptor (AT1R) mRNA (**A**) and angiotensinogen mRNA (**B**) expression in WAT and BAT of ATRAP^fl/fl^ and ATRAP^adipoq^ mice fed an LFD (*n* = 5–6). Values are the means ± standard error of the mean (SEM). AT1R: Angiotensin II type1 receptor; WAT: White adipose tissue; BAT: Brown adipose tissue; LFD: Low-fat diet.

To study the effect of ATRAP deletion in adipose tissue, we generated an *Agtrap* (angiotensin II receptor associated protein) gene “floxed” mouse (*Agtrap^fl/fl^*) strain carrying the *loxP*-flanked *Agtrap* allele. *Agtrap^fl/fl^* mice were crossed with *Adipoq-Cre* transgenic mice that expressing Cre recombinase under control of the mouse adiponectin promoter [18]. The resulting *Agtrap^fl/fl^*/*Adipoq-Cre* mice were then mated with *Agtrap^fl/fl^* mice to generate *Agtrap^fl/fl^*/*Adipoq-Cre* (ATRAP^adipoq^) mice and control *Agtrap^fl/fl^* mice without *Adipoq-Cre* (ATRAP^fl/fl^). Figure 1A shows the strategy of conditional gene targeting for *Agtrap*. To verify the presence of *loxP* sites within the *Agtrap* gene of the generated mice, tail DNAs were analyzed by PCR amplification for the first *loxP* region in intron 2 and the second *loxP* region in intron 4. Similarly, *Cre* transgene was identified by PCR genotyping (Figure 1B). To confirm the efficiency and specificity of ATRAP downregulation in adipose tissue, we quantified the ATRAP mRNA abundance in each tissue from ATRAP^adipoq^ and ATRAP^fl/fl^ mice fed a low-fat diet (LFD) by real-time reverse transcription quantitative polymerase chain reaction (RT-qPCR) analysis. ATRAP^adipoq^ mice expressed approximately a 30% reduction of ATRAP mRNA in the WAT and approximately an 85% reduction in the BAT compared with ATRAP^fl/fl^ mice, but no significant change in other tissues (Figure 1C). In addition, we examined ATRAP protein levels in the WAT and BAT from ATRAP^adipoq^ and ATRAP^fl/fl^ mice fed an LFD. Similarly, while ATRAP^adipoq^ mice exhibited a mild decrease in protein levels of ATRAP expression in the WAT compared with ATRAP^fl/fl^ mice, these mice exhibited that a marked decrease in protein levels of ATRAP expression is the BAT (Figure 1D). Since ATRAP is associated with the deactivation of AT1R signaling, we further examined whether expression of other RAS components such as angiotensinogen and AT1R would be altered in the WAT and BAT of ATRAP^adipoq^ mice. As shown in Figure 2, the mRNA expression levels of angiotensinogen and AT1R in the WAT and BAT of ATRAP^adipoq^ mice were comparable with those in the WAT and BAT of ATRAP^fl/fl^ mice.

### 2.2. Physiological and Metabolic Status of ATRAP^adipoq^ Mice

#### 2.2.1. Body Weight Changes and Physiologic Parameters

**Figure 3:**
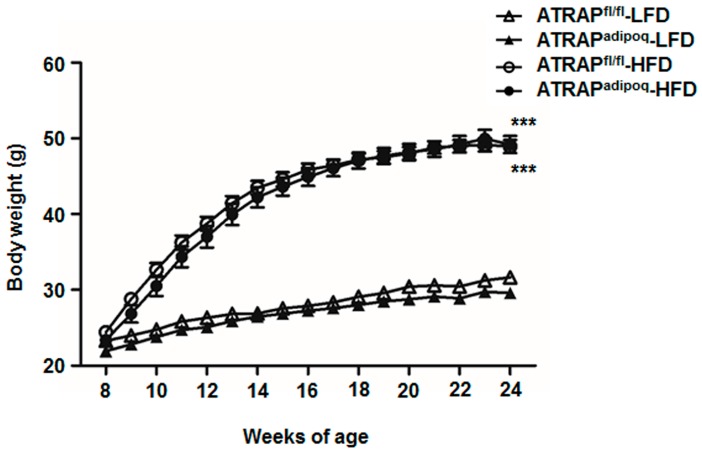
Change in body weight in ATRAP^fl/fl^ and ATRAP^adipoq^ mice fed an LFD or HFD (*n* = 9). ∆, ATRAP^fl/fl^ mice fed an LFD; ▲, ATRAP^adipoq^ mice fed an LFD; ○, ATRAP^fl/fl^ mice fed an HFD; ●, ATRAP^adipoq^ mice fed an HFD. Values are the means ± SEM. *** *p* < 0.001 vs. LFD within the same group. Data were analyzed by two-way repeated measures analysis of variance (ANOVA). LFD: Low-fat diet; HFD: High-fat diet.

At baseline, there were no significant differences in body weight, systolic blood pressure, heart rate, rectal temperature, tissue weight, and plasma physiological parameters between the ATRAP^fl/fl^ and ATRAP^adipoq^ mice on the LFD (Table 2). High-fat diet loading for 16 weeks significantly increased body weight in both genotypes to similar degrees (Figure 3). The WAT, BAT, and liver weight were similarly increased by an HFD in both types of mice (Table 2). The systolic blood pressure (SBP) of ATRAP^fl/fl^ mice was significantly increased by an HFD. ATRAP^adipoq^ mice had no significant increase of SBP by HFD, which was similar to the SBP of ATRAP^fl/fl^ mice fed an HFD. Plasma total cholesterol and insulin concentrations were significantly increased by HFD in both types of mice. However, there were no significant differences between the two genotypes (Table 2). Although only ATRAP^adipoq^ mice had an increased plasma glucose concentration by HFD, there was no significant difference between genotypes on either diet (Table 2).

#### 2.2.2. Glucose and Insulin Tolerance

**Figure 4:**
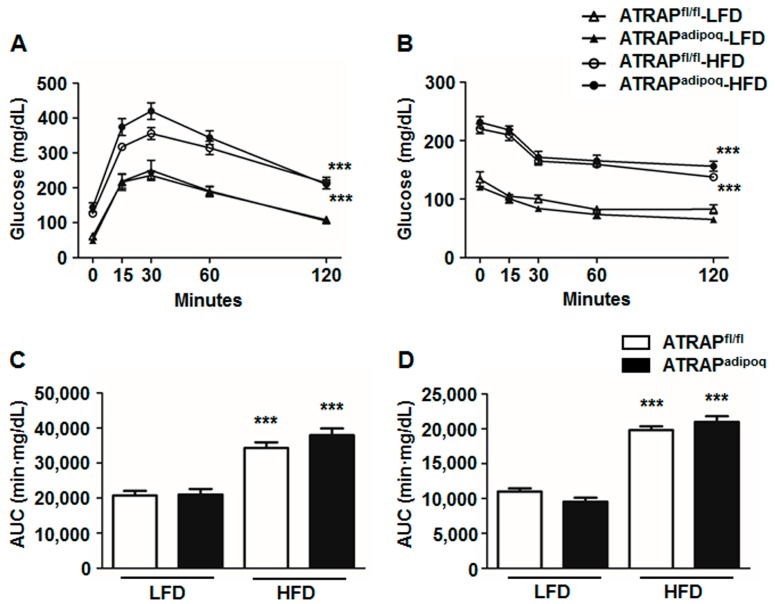
Glucose tolerance test (GTT) and insulin tolerance test (ITT). (**A**,**B**) The GTT and ITT in ATRAP^fl/fl^ and ATRAP^adipoq^ mice after 16-week LFD or HFD feeding (*n* = 5–15). ∆, ATRAP^fl/fl^ mice fed an LFD; ▲, ATRAP^adipoq^ mice fed an LFD; ○, ATRAP^fl/fl^ mice fed an HFD; ●, ATRAP^adipoq^ mice fed an HFD; (**C**,**D**) The area under the curve (AUC) of GTT and ITT in ATRAP^fl/fl^ and ATRAP^adipoq^ mice after 16-week LFD or HFD feeding (*n* = 5–15). Values are the means ± SEM. (**A**,**B**) *** *p* < 0.001 vs. LFD within the same group. Data were analyzed by two-way repeated measures ANOVA. (**C**,**D**) *** *p* < 0.001 vs. LFD within the same group. Data were analyzed by two-way ANOVA. LFD: Low-fat diet; HFD: High-fat diet.

To examine the effects of adipose ATRAP downregulation on insulin resistance, we performed a glucose tolerance test (GTT) and an insulin tolerance test (ITT), which reflect the glucose tolerance and insulin sensitivity, respectively. There were no significant differences in GTT between the ATRAP^fl/fl^ and ATRAP^adipoq^ mice fed an LFD. High-fat diet loading for 16 weeks significantly exacerbated glucose intolerance in the ATRAP^fl/fl^ and ATRAP^adipoq^ mice. However, there were no significant differences in glucose intolerance between the genotypes (Figure 4A,C). Similarly, the ITT showed that HFD significantly exacerbated insulin resistance in both types of mice to similar degrees (Figure 4B,D).

### 2.3. Effects of High-Fat Diet on Adipocyte Hypertrophy and Macrophage Infiltration in ATRAP^fl/fl^ and ATRAP^adipoq^ Mice

**Figure 5:**
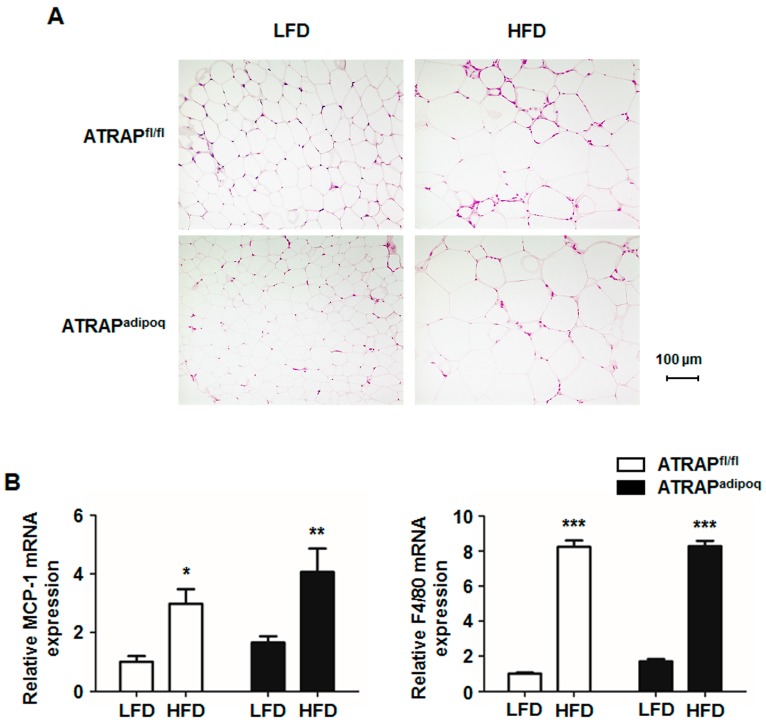
Adipocyte morphology and inflammation of WAT. (**A**) Representative images of adipocyte cells in WAT of ATRAP^fl/fl^ and ATRAP^adipoq^ mice fed an LFD and HFD. (**B**) Relative monocyte chemotactic protein-1 (MCP-1) and F4/80 mRNA expression in WAT of ATRAP^fl/fl^ and ATRAP^adipoq^ mice fed an LFD and HFD (*n* = 5–6). Values are the means ± SEM. * *p* < 0.05, ** *p* < 0.01, *** *p* < 0.001 vs. LFD within the same group. Data were analyzed by two-way ANOVA. LFD: Low-fat diet; HFD: High-fat diet.

We next examined adipocyte morphology and inflammation in ATRAP^fl/fl^ and ATRAP^adipoq^ mice on LFD and HFD. The results of histological analysis showed that there were no differences in WAT adipocyte morphology between the ATRAP^fl/fl^ and ATRAP^adipoq^ mice fed an LFD. High-fat diet loading for 16 weeks caused adipocyte hypertropy in both types of mice, to the same extent (Figure 5A). In addition, we examined inflammation-related gene expressions in WAT of ATRAP^fl/fl^ and ATRAP^adipoq^ mice. High-fat diet loading for 16 weeks significantly increased mRNA expression of F4/80 and monocyte chemotactic protein-1 (MCP-1), markers of macrophage infiltration, in WAT. However, there were no significant differences in the upregulation of these genes in response to HFD between the genotypes (Figure 5B).

### 2.4. White Adipose Tissue ATRAP mRNA Expression in ATRAP^adipoq^ Mice Is Comparable to ATRAP^fl/fl^ Mice on a High-Fat Diet

**Figure 6:**
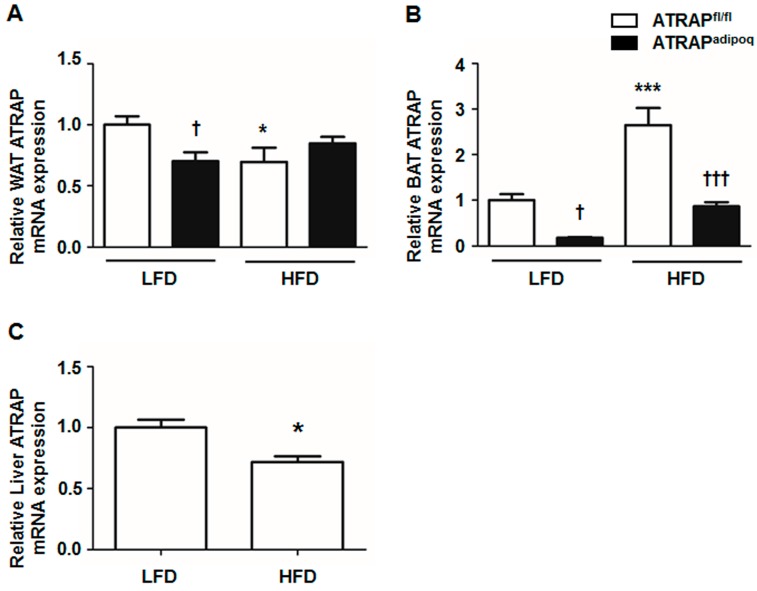
The changes of ATRAP expression in adipose tissues and liver with HFD. (**A**) Relative WAT ATRAP mRNA expression in ATRAP^fl/fl^ and ATRAP^adipoq^ mice fed an LFD and HFD (*n* = 6). (**B**) Relative BAT ATRAP mRNA expression in ATRAP^fl/fl^ and ATRAP^adipoq^ mice fed an LFD and HFD (*n* = 6). Relative liver ATRAP mRNA expression in ATRAP^fl/fl^ mice fed an LFD and HFD (*n* = 6). Values are the means ± SEM. (A, B) ^†^ *p* < 0.05, ^†††^ *p* < 0.001 vs. ATRAP^fl/fl^ mice on the same diet. * *p* < 0.05, *** *p* < 0.001 vs. LFD within the same group. Data were analyzed by two-way ANOVA. (**C**) * *p* < 0.05 vs. LFD group. Data were analyzed by an unpaired Student’s *t*-test. LFD: Low-fat diet; HFD: High-fat diet.

Because there were no differences in physiological and metabolic parameters, adipocyte morphology, and adipose macrophage infiltration between the ATRAP^fl/fl^ and ATRAP^adipoq^ mice on HFD or LFD, we examined the ATRAP mRNA expression in the WAT. As shown in Figure 6A, ATRAP mRNA levels in the WAT from ATRAP^adipoq^ mice were reduced by about 30% compared with ATRAP^fl/fl^ mice on LFD similar to that observed for the tissue distribution of ATRAP mRNA expression (Figure 1C). High-fat diet for 16 weeks significantly decreased WAT ATRAP mRNA expression in ATRAP^fl/fl^ mice, but not ATRAP^adipoq^ mice. There were no significant differences in WAT ATRAP mRNA expression between ATRAP^fl/fl^ and ATRAP^adipoq^ mice fed an HFD (Figure 6A). In contrast, ATRAP mRNA levels in the BAT from ATRAP^adipoq^ mice were markedly decreased compared with ATRAP^fl/fl^ mice either on LFD or HFD (Figure 6B). In addition, we examined whether endogenous ATRAP mRNA expression is changed by HFD loading in tissues other than adipose tissue. In liver, HFD for 16 weeks significantly decreased ATRAP mRNA expression in ATRAP^fl/fl^ mice (Figure 6C).

## 3. Discussion

Six major findings were obtained in our study: (i) the rate of decrease in ATRAP mRNA in ATRAP^adipoq^ mice generated using the *Adipoq-Cre* line with the Cre/loxP system was approximately 30% in the WAT and 85% in the BAT fed an LFD; (ii) an HFD significantly decreased endogenous ATRAP expression in the WAT and liver in *Agtrap*-floxed mice, concomitant with an increase in dietary obesity and insulin resistance; (iii) ATRAP expression in the WAT was similar between ATRAP^adipoq^ and ATRAP^fl/fl^ mice when fed an HFD; (iv) ATRAP expression in the BAT from ATRAP^adipoq^ mice was markedly decreased compared with ATRAP^fl/fl^ mice even when fed an HFD; (v) no significant difference was observed in glucose and lipid metabolism in ATRAP^adipoq^ and ATRAP^fl/fl^ mice fed an HFD; and (vi) ATRAP expression in the BAT did not appear to influence the pathogenesis of obesity-related metabolic disorders.

We used the *Adipoq-Cre* line to generate mice in which target genes were knocked out specifically in adipose tissues [18]. The *aP2-Cre* line created by multiple laboratories has had a major role in the generation of mouse models in which adipose tissue-specific genes were knocked out. However, problems such as the endogenous expression of fatty acid binding protein 4 (FABP4) in macrophages, vascular endothelial cells, and ectopic Cre expression in blood cells, heart, and skeletal muscle, have been identified [19,20,21]. In contrast, the *Adipoq-Cre* line was reported to be superior to the *aP2-Cre* line regarding adipocyte specificity [22,23]. Therefore, we chose the *Adipoq-Cre* line to localize Cre-dependent gene recombination to the adipose tissue (adipocytes). However, the decrease in ATRAP mRNA expression in the WAT of ATRAP^adipoq^ mice was unexpectedly mild. Recombination sensitivity mediated by Cre, which differs substantially depending on the target gene locus, was likely one of the reasons for this mild decrease [22,24,25]. Similar to our study, several gene-modified models generated using the *Adipoq-Cre* line showed poor recombination efficiency in the WAT compared with the BAT [22]. Indeed, recombination efficiency is dependent on adipose tissue type, e.g., perigonadal adipose tissue (visceral fat tissue), subcutaneous adipose tissue, and BAT. In addition, other non-adipocyte cells in adipose tissue might express high levels of ATRAP, such as macrophages. Therefore, further studies will be needed to compare ATRAP expression in adipocytes and stromal vascular fractions isolated from WAT between ATRAP^adipoq^ and ATRAP^fl/fl^ mice.

ATRAP selectively prevents the excessive activation of the AT1R signaling pathway by various pathological stimuli. We previously reported the in vivo regulation of ATRAP expression. The relative expression of ATRAP to AT1R in the heart of spontaneously hypertensive rats (SHRs) was decreased as hypertension and cardiac hypertrophy progressed [26]. In Dahl salt-sensitive rats, high-salt diet loading decreased renal ATRAP expression and accelerated the progression of hypertensive kidney injury [27]. Expression of ATRAP in the WAT was decreased in KKAy mice, a diabetes mellitus model, compared with control C57BL/6N mice [16]. We also reported changes in tissue ATRAP expression in response to a pathological stimulus. For example, chronic Ang II infusion in mice decreased ATRAP expression in the kidney and heart and accelerated the progression of hypertension and cardiac hypertrophy [28,29]. In mice that underwent unilateral ureteral obstruction, renal ATRAP expression was decreased as renal fibrosis progressed [30]. In the present study, we determined that HFD loading in ATRAP^fl/fl^ mice decreased endogenous ATRAP expression in the WAT and exacerbated the progression of dietary obesity and insulin resistance. This finding suggests that endogenous ATRAP alone is likely insufficient to suppress lifestyle diseases, such as metabolic syndrome, because its expression is decreased by pathological stimuli.

ATRAP^adipoq^ mice showed a phenotype similar to that of ATRAP^f1/f1^ mice fed an LFD or HFD. Because ATRAP does not modulate the physiological AT1R signaling pathway in the absence of pathological stimuli, no clear difference was expected between genotypes fed an LFD [16]. However, even dietary obesity induced by an HFD did not result in differences in glucose or lipid metabolism between ATRAP^adipoq^ and ATRAP^f1/f1^ mice. This finding can be explained by the fact that HFD loading on ATRAP^fl/fl^ mice decreased the endogenous ATRAP expression level in the WAT, thus negating the difference in ATRAP expression levels between the genotypes. ATRAP function in the WAT of ATRAP^adipoq^ mice is likely to be similar to that in ATRAP^f1/f1^ mice because of the similar Expression levels of ATRAP mRNA in the WAT between the genotypes when fed an HFD. Moreover, dietary obesity altered the WAT composition—adipocytes and stromal vascular cells (SVC) including macrophages—and induced angiogenesis [9]. In fact, HFD loading caused adipocyte hypertrophy and macrophage infiltration in WAT of mice in the present study. However, recombination by *Adipoq-Cre* specifically targets adipocytes, but not SVC [21,22]. In this regard, further studies will be needed to investigate ATRAP expression in adipocytes and SVC isolated from WAT.

Brown adipose tissue acts antagonistically on dietary obesity by promoting thermogenesis, mainly by uncoupling protein-1 production, which, in turn, accelerates energy metabolism [31]. In the present study, ATRAP expression in the BAT was markedly decreased in ATRAP^adipoq^ mice compared with ATRAP^fl/fl^ mice. We also showed that ATRAP expression in BAT was significantly increased by HFD feeding in mice. Although the reasons for increasing ATRAP expression in BAT by HFD feeding are unclear at present, importantly, ATRAP^adipoq^ mice still exhibited a decreased level of ATRAP expression in BAT compared with ATRAP^fl/fl^ mice when fed an HFD. Nevertheless, the rectal temperature was comparable between the two genotypes fed an LFD and an HFD. This result is not surprising considering the very low levels of ATRAP in BAT compared to WAT.

To further study in vivo ATRAP function in the WAT in terms of dietary obesity, our results suggest the use of a mouse model other than the adipose tissue-specific *Cre* line and changing the duration of diet loading. The candidate lines include *adiponectin-CreERT* (tamoxifen-inducible Cre) [21], an *adiponectin-Cre* line generated by Scherer et al. [32] with a high efficiency in Cre recombination, conventional *aP2-Cre*, and *Retn-Cre*—using a 33 kb fragment of the resistin (*Retn*) gene—recently reported by the laboratory of Lazar et al. [33]. Because HFD loading decreased endogenous ATRAP expression in the WAT of ATRAP^fl/fl^ mice, studies using an adipose tissue-specific ATRAP overexpression model are thought to be a useful study tool. Recently, we reported that adipose tissue-specific ATRAP transgenic mice exhibit a suppression of HFD-induced visceral obesity and insulin resistance [34]. Since the establishment of conditional knockouts using the Cre/loxP system, numerous studies have been published, indicating the importance and convenience of this approach. However, as noted in our study, experimental conditions such as diet can influence the characteristics of these animal models. Thus, researchers should be careful in their interpretation of results in this field.

## 4. Materials and Methods

### 4.1. Animals and Animal Care

This study was performed in accordance with the National Institutes of Health (NIH) “Guide for the Care and Use of Laboratory Animals.” All animal studies were reviewed and approved by the Animal Studies Committee of Yokohama City University (Yokohama, Japan). The protocol code is FA-16-025 approved at March 31, 2016. The mice were housed in a controlled environment with a 12 h light-dark cycle at a temperature of 25 °C and were allowed free access to food and water. They were fed either an LFD (3.6 kcal/g; 13.3% energy as fat; The Oriental Yeast Co., Ltd. Tokyo, Japan) or HFD (5.6 kcal/g; 60.0% energy as fat) for 16 weeks beginning at 8 weeks of age, and their body weights were measured weekly. All experiments in this study were performed with *Adipoq-Cre^+^/Agtrap^fl/fl^* mice and littermate *Agtrap^fl/fl^* mice. At the end of the experimental period (24 weeks of age), mice were anesthetized with an intraperitoneal injection of pentobarbital and sacrificed in the fed state between 10:00 a.m.–2:00 p.m.

### 4.2. Generation of Adipoq-Cre^+^/Agtrap^fl/fl^ Mice

#### 4.2.1. Generation of *Agtrap^fl/fl^* Mice

**Figure 7:**
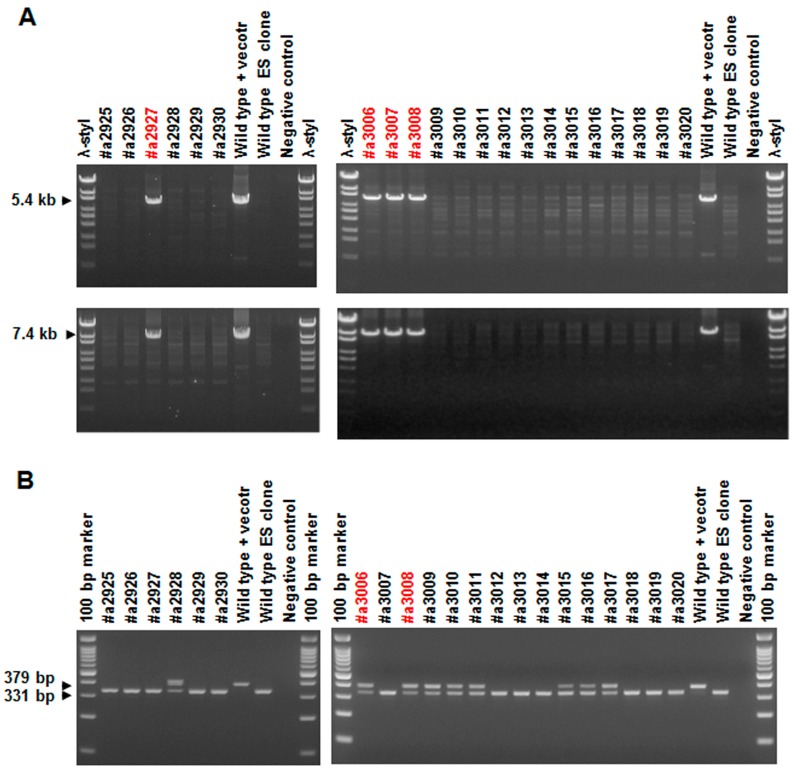
Agarose-gel electrophoresis of PCR products from embryonic stem (ES) cell clones’ DNA (#a2925–a2930, #a3006–a3020), amplified for homologous recombination (**A**) and for detecting second *loxP* (**B**). (**A**) 5′ PCR using primer set: 5AF4/neo100 (upper panel) and 3′ PCR using primer set: neo marker sense/3AR2 (lower panel); red-labeled clones were positive for amplification. (**B**) PCR using primer set: F24587/R2491; red-labeled clones were positive for amplification of mutant *loxP* within positive for homologous recombination.

**Figure 8:**
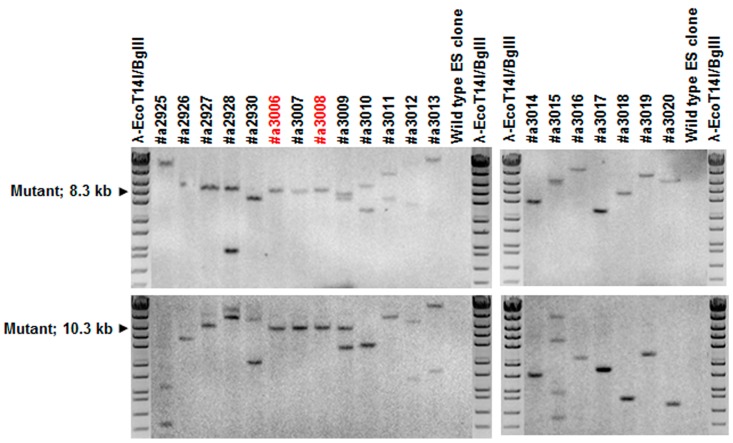
Southern blot analysis using neo probe. 5′ EcoRI-digested (target mutant band is 8.3 kb in upper panel) and 3′ NdeI-digested (target mutant band is 10.3 kb in lower panel) DNA were analyzed. #a3006 and #a3008 clones (in red) were verified to have one inserted copy of the targeting vector.

**Figure 9:**
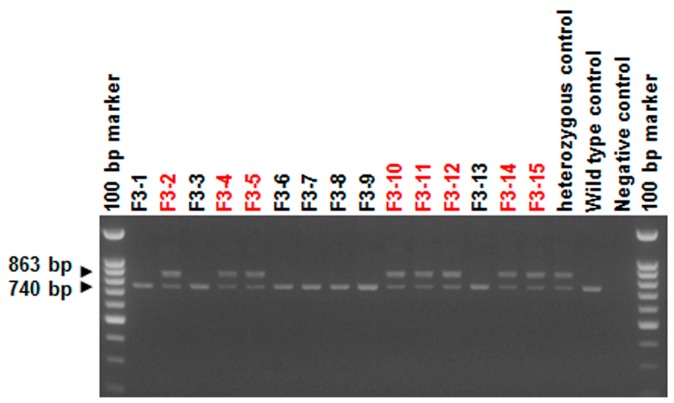
Agarose-gel electrophoresis of PCR products amplified to detect first *loxP* using the primer set F23005 and R23744. Analyzed DNAs (F3-1–F3-15) were from F3 mice with heterozygous *Agtrap* floxed allele from which the *PGK-Neo* cassette was removed. The red-labeled sample were positive for *loxP* site amplification.

For conditional *Agtrap* gene targeting, we generated an *Agtrap* gene “floxed” mouse where two *loxP* sequences were inserted into the mouse *Agtrap* locus by homologous recombination (Figure 1A). This series of processes was performed by TransGenic Inc. (Fukuoka, Japan). To construct the targeting vector, a 4.2 kb mouse genomic fragment containing intron 1, exon 2 and intron 2, a 1.1 kb fragment containing intron 2, exon 3, intron 3, exon 4 and intron 4, and a 5.7 kb fragment containing intron 4, exon 5 and the 3′ region of the *Agtrap* gene were amplified by PCR from RENKA embryonic stem (ES) cell genomic DNA, the cell line was established by the laboratory of Sakimura et al. (Niigata, Japan) [35]. The 4.2 kb genomic fragment was cloned into a plasmid vector containing *loxP* sequences, a *PGK-Neo* cassette (phosphoglycerate kinase 1 promoter driven neomycin resistant gene) flanked by *FRT* sequences as a positive selection marker, and a *PGK-TK* cassette (phosphoglycerate kinase 1 promoter driven SV40 thymidine kinase gene) as a negative selection marker. Then, the 1.1 kb genomic fragment was subcloned into the plasmid. Subsequently, a 5.7 kb genomic fragment was inserted into the plasmid. The resulting targeting vector contained a *PGK-TK* cassette, a 4.2 kb 5′ homologous arm, first *loxP* site, *FRT*-flanked *PGK-Neo* cassette, 1.1 kb-floxed genomic region containing exons 3 and 4 of the *Agtrap* gene, second *loxP* site, and 5.7 kb 3′ homologous arm. This targeting vector was linearized and introduced into RENKA ES cells (C57BL/6N) by electroporation. After selection using Geneticin, the resistant clones were isolated, and their DNAs were screened for homologous recombinant by nested PCR using the following primer sets: 5AF5 and neo108r for first amplification, and 5AF4 and neo100 for second amplification of nested PCR. Homologous recombinations (PCR-positive ES clone) were confirmed in four clones (#a2927, #a3006, #a3007, #a3008). PCR-positive ES clones were expanded, and isolated DNAs were further analyzed by PCR amplification using the following primer sets: 5AF4 and neo100 for 5′ amplification, neo marker sense and 3AR2 for 3′ amplification (Figure 7A), and F24587 and R24917 for amplification of the second *loxP* region (Figure 7B). There were two clones with *loxP* sites (#a3006, a3008). Homologous recombination of these clones was confirmed by genomic Southern hybridization probed with a neomycin resistant gene. The predicted size was detected (Figure 8). Homologous recombinant ES cell clones (#a3006, #a3008) were aggregated with ICR 8 cell embryos to generate chimeric mice. Germline transmitted F1 heterozygous mice were obtained by crossing chimeric mice with a high contribution of the RENKA background with C57BL/6N mice. The targeted allele was identified by PCR with the following primer set: 5AF4 and neo100. To remove the *PGK-Neo* cassette, F1 heterozygous mice were crossed with B6; D2-*Tg*(*CAG-Flp*)*18Imeg* (obtained from the Center for Animal Resources and Development (CARD), Kumamoto, Japan) transgenic mice, which express Flp recombinase in germ cells. Floxed allele without a *PGK-Neo* cassette was identified with the following PCR primer set: F23005 and R23744 (Figure 9). The primer sequences are shown in Table 1. Obtained heterozygous floxed (*Agtrap*^fl/-^) mice were further mated with *Agtrap*^fl/-^ mice to generate homozygous floxed (*Agtrap^fl/fl^*) mice.

#### 4.2.2. Generation of *Adipoq-Cre^+^/Agtrap^fl/fl^* Mice

To inactivate the *Agtrap* gene in adipocytes, *Agtrap^fl/fl^* mice were intercrossed with *Adipoq-Cre* transgenic mice expressing Cre recombinase under control of the mouse adiponectin promoter [18]. *Adipoq-Cre* transgenic mice were obtained from the Jackson Laboratory (Bar Harbor, Maine, USA). The resulting *Adipoq-Cre^+^/ Agtrap^fl/fl^* mice were mated with *Agtrap^fl/fl^* mice to generate *Adipoq-Cre^+^/Agtrap^fl/fl^* mice (ATRAP^adipoq^) and control *Agtrap^fl/fl^* mice without *Adipoq-Cre* (ATRAP^fl/fl^). 

### 4.3. Blood Pressure Measurement by the Tail–Cuff Method

Systolic blood pressure and heart rate were measured by the tail-cuff method (BP-monitor MK-2000; Muromachi Kikai Co., Ltd., Tokyo, Japan) such that the blood pressure was measured without any preheating of the animals, as described previously [26,36]. All measurements were performed between 10:00 a.m.–2:00 p.m., with at least eight values taken for each measurement.

### 4.4. Biochemical Assay

Blood samples were obtained by cardiac puncture when the mice were sacrificed in the fed state. Whole blood samples were centrifuged at 3000 rpm (MR-150, Tomy Seiko Co., Ltd., Tokyo, Japan) at 4 °C for 15 min to separate the plasma. The resulting plasma was stored at −80 °C until use. Enzymatic assays were used to determine the plasma glucose, total cholesterol, triglycerides, and non-esterified fatty acids (Wako Pure Chemical, Osaka, Japan). The plasma insulin concentration was measured with a commercially available enzyme-linked immunosorbent assay (ELISA) kit (Morinaga Institute of Biological Science, Inc., Yokohama, Japan).

### 4.5. Glucose and Insulin Tolerance Tests

The GTT and ITT were performed as previously described with slight modifications [16]. Briefly, GTT and ITT were performed 4 days apart at the end of the experimental period between 10:00 a.m.–2:00 p.m. and 2:00 p.m.–4:00 p.m., respectively. For GTT, blood glucose concentrations were measured with a blood glucose test meter (Glutest Neo Super; Sanwa-Kagaku Co., Ltd., Nagoya, Japan) using blood samples taken from the tail tip of overnight-fasted mice at baseline and at 15, 30, 60 and 120 min after the intraperitoneal injection of glucose (1 g/kg body weight). For ITT, insulin (0.75 U/kg body weight in 0.1% bovine serum albumin (BSA); Humulin R-Insulin; Eli Lilly and Co., Indiana, IN, USA) was administered via intraperitoneal injection after 4-h fasting. Blood glucose concentrations were measured at baseline and at 15, 30, 60 and 120 min after the injection.

### 4.6. Real-Time RT-qPCR Analysis

Total RNAs were extracted from the white adipose tissue with ISOGEN (Nippon Gene, Toyama, Japan) and the cDNA was synthesized using the SuperScript III First-Strand System (Invitrogen, Carlsbad, CA, USA). Real-time RT-qPCR was performed with an ABI PRISM 7000 Sequence Detection System (Applied Biosystems, Foster, CA, USA) by incubating the reverse transcription product with TaqMan PCR Master Mix and a designed Taqman probe (Applied Biosystems, Foster, CA, USA), as described previously [29]. The mRNA levels were normalized to those of the glyceraldehyde 3-phosphate dehydrogenase (GAPDH) control.

### 4.7. Western Blot Analysis

Western blot analysis was performed as described previously [28]. Briefly, total protein was extracted from WAT with a sodium dodecyl sulfate (SDS)-containing sample buffer. Then, the protein concentration of each sample was measured with a detergent compatible (DC) protein assay kit (Bio-Rad, Hercules, CA, USA) using bovine serum albumin as the standard. Equal amounts of protein extract were fractionated on a 5%–20% polyacrylamide gel (ATTO) and then transferred to a polyvinylidene difluoride (PVDF) membrane using the iBlot Dry Blotting System (Invitrogen). Membranes were blocked for 1 h at room temperature with phosphate-buffered saline (PBS) containing 5% skim milk powder and probed overnight at 4 °C with specific primary antibodies to ATRAP.

### 4.8. Histological Analysis

The epididymal WAT was collected and fixed with 10% paraformaldehyde overnight and embedded in paraffin. Tissue sections were stained with hematoxylin and eosin as described previously [16]. All images were acquired using a BZ-9000 microscope (Keyence, Osaka, Japan).

### 4.9. Statistical Analysis

All data are shown as the mean ± standard error of the mean (SEM). Differences were analyzed as follows. A two-way analysis of variance (ANOVA) with a Bonferroni post-test was used to test for differences in diet within each genotype or differences in genotype for mice on the same diet. A two-way repeated measure ANOVA was used to test for differences over time. An unpaired Student’s *t*-test was used to test for differences between two groups.

## 5. Conclusions

In conclusion, we report that wild-type mice fed an HFD showed decreased endogenous ATRAP expression in the WAT concomitant with the enhanced progression of dietary obesity and insulin resistance. Expression of ATRAP mRNA in the BAT was decreased by approximately 85% in mice with suppressed adipose tissue-specific ATRAP expression generated with the Cre/loxP system and the *Adipoq-Cre* line, compared with *Agtrap*-floxed control mice. Expression of ATRAP mRNA in the WAT of *Agtrap* gene-modified mice was decreased by approximately 30% at baseline of LFD but was similar to control mice after HFD loading. No significant difference between genotypes was detected regarding glucose and lipid metabolism after HFD loading. Overall, the results of the present study indicate that ATRAP expression in BAT does not influence the development of HFD-induced metabolic disorders.

## Figures and Tables

**Table 1:** Primer sequences to generate *Agtrap^fl/fl^* and *Adipoq-Cre^+^/Agtrap^fl/fl^* mice.

Primer	Sequence (5′–3′)	Target Region
F23005	CCTCTTCTGGACCACTCTATCTCTCTGC	first *loxP*
R23744	GTTCCAGGGTCTTAACCTCCTCTGAG	
F24587	CTCGTCTACCACATGCACCGTCAACG	second *loxP*
R24917	AGCTCCCATAGAATAGGTTCAGAGAGG	
cre-fw	AGGTTCGTGCACTCATGGA	*cre*
cre-rw	TCGACCAGTTTAGTTACCC	
mAgtrapChIPF	CCTAGCAGCAAGAGCAGCT	*agtrap*
mAgtrapChIPR	GAACTCGGGAACAAACTTCCT	
5AF5	AGTGAATTCATTATCTAGGGACAGAATTACAGG	5′-targeted allele (1st)
neo108r	CCTCAGAAGAACTCGTCAAGAAG	
5AF4	AGTGAATTCAGGTCAGGCTCTGCCTTATTCTGC	5′-targeted allele (nested)
neo100	AGGTGAGATGACAGGAGATC	
neo_marker_sense	ATTCGCAGCGCATCGCCTTCTATCGCCTTC	3′-targeted allele
3AR2	TAAGCGGCCGCTCTCCCCAGAAATAGCTGGAAATCACC	

**Table 2:** Physiological and metabolic parameters of mice on a low-fat diet or high-fat diet.

Variables	LFD		HFD	
	ATRAP^fl/fl^	ATRAP^adipoq^	ATRAP^fl/fl^	ATRAP^adipoq^
Body weight (g)	31.6 ± 0.6	29.5 ± 0.5	48.9 ± 0.8 ***	49.1 ± 1.1 ***
Systolic blood pressure (mmHg)	114 ± 1	116 ± 1	124 ± 2 *	124 ± 3
Heart rate (beat/min)	742 ± 1	747 ± 15	712 ± 8	743 ± 11
Rectal temperature (°C)	37.2 ± 0.2	37.8 ± 0.3	37.8 ± 0.1	37.7 ± 0.1
Tissue weight				
Epididymal adipose tissue (mg)	872 ± 160	694 ± 144	1293 ± 25 ***	1220 ± 48 ***
Brown adipose tissue (mg)	139 ± 14	128 ± 7	425 ± 27 ***	404 ± 22 ***
Liver (mg)	1510 ± 127	1407 ± 50	3670 ± 226 ***	3242 ± 192 ***
Heart (mg)	148 ± 9	139 ± 7	156 ± 6	145 ± 4
Plasma concentration				
Total cholesterol (mg/dL)	83 ± 8	91 ± 6	250 ± 16 ***	218 ± 12 ***
Triglyceride (mg/dL)	35 ± 9	38 ± 8	35 ± 4	22 ± 1
Non-esterified fatty acid (µEq/L)	470 ± 147	496 ± 143	456 ± 94	241 ± 42
Glucose (mg/dL)	232 ± 16	206 ± 11	259 ± 19	288 ± 25 *
Insulin (ng/mL)	1.3 ± 0.2	1.3 ± 0.1	8.1 ± 1.7 **	7.1 ± 2.2 *

All of the values are the means ± SEM. * *p* < 0.05, ** *p* < 0.01, *** *p* < 0.001 vs. LFD within the same group. *n* = 6–12 per group. Data were analyzed by two-way ANOVA. ATRAP^fl/fl^: *Agtrap*-floxed control mice; ATRAP^adipoq^: *Agtrap*-floxed with *Adipoq-Cre* transgenic mice; LFD: Low-fat diet; HFD: High-fat diet.

## Abbreviations

*Adipoq*	Adiponectin gene
*Agtrap*	ATRAP gene
AT1R	Angiotensin II type 1 receptor
ATRAP	Angiotensin II type 1 receptor associated protein
BAT	Brown adipose tissue
GTT	Glucose tolerance test
HFD	High-fat diet
ITT	Insulin tolerance test
LFD	Low-fat diet
MCP-1	Monocyte chemotactic protein-1
WAT	White adipose tissue
